# Supplementary material for: Isolation of intact extracellular vesicles from cryopreserved samples
Source: PLoS One. 2021 May 13;16(5):e0251290. doi: 10.1371/journal.pone.0251290 (PMC8118530; doi:10.1371/journal.pone.0251290)
Supplement: S1 Fig — All plasma samples were added directly to the -80°C freezer (without a freezing container), thawed at room temperature, and isolated using ultracentrifugation. (A-D) Particle rate expressed as line graphs of fresh versus stored samples are illustrated with a black or red line, respectively, for representative donors. A higher particle rate indicates samples have a higher concentration of EVs. (DOCX) [file pone.0251290.s001.docx]

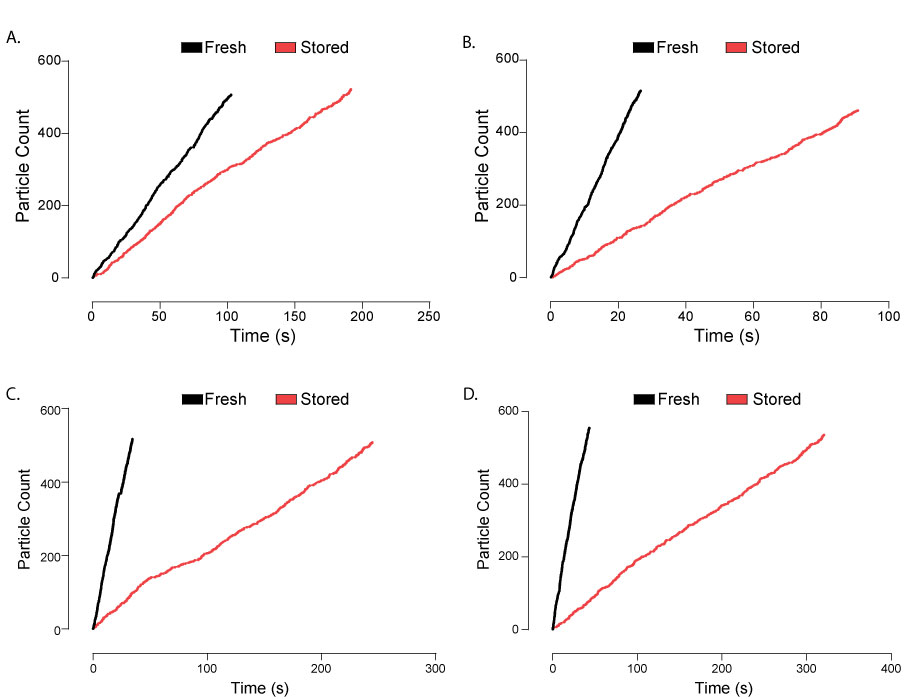


**Supplementary Figure 1**: *Impact of cryopreservation on particle rate of plasma-derived EVs*. All plasma samples were added directly to the -80°C freezer (without a freezing container), thawed at room temperature, and isolated using ultracentrifugation. (A-D) Particle rate expressed as line graphs of fresh versus stored samples are illustrated with a black or red line, respectively, for representative donors. A higher particle rate indicates samples have a higher concentration of EVs.
