# Supplementary material for: Isolation of intact extracellular vesicles from cryopreserved samples
Source: PLoS One. 2021 May 13;16(5):e0251290. doi: 10.1371/journal.pone.0251290 (PMC8118530; doi:10.1371/journal.pone.0251290)
Supplement: S2 Fig — All plasma samples were added directly to the -80°C freezer (without a freezing container), thawed at room temperature, and isolated using ultracentrifugation. (A-B) Representative histogram for two donors showing EV concentration binned by particle diameter (nm) with fresh samples shown in dark grey and stored samples in red. (DOCX) [file pone.0251290.s002.docx]

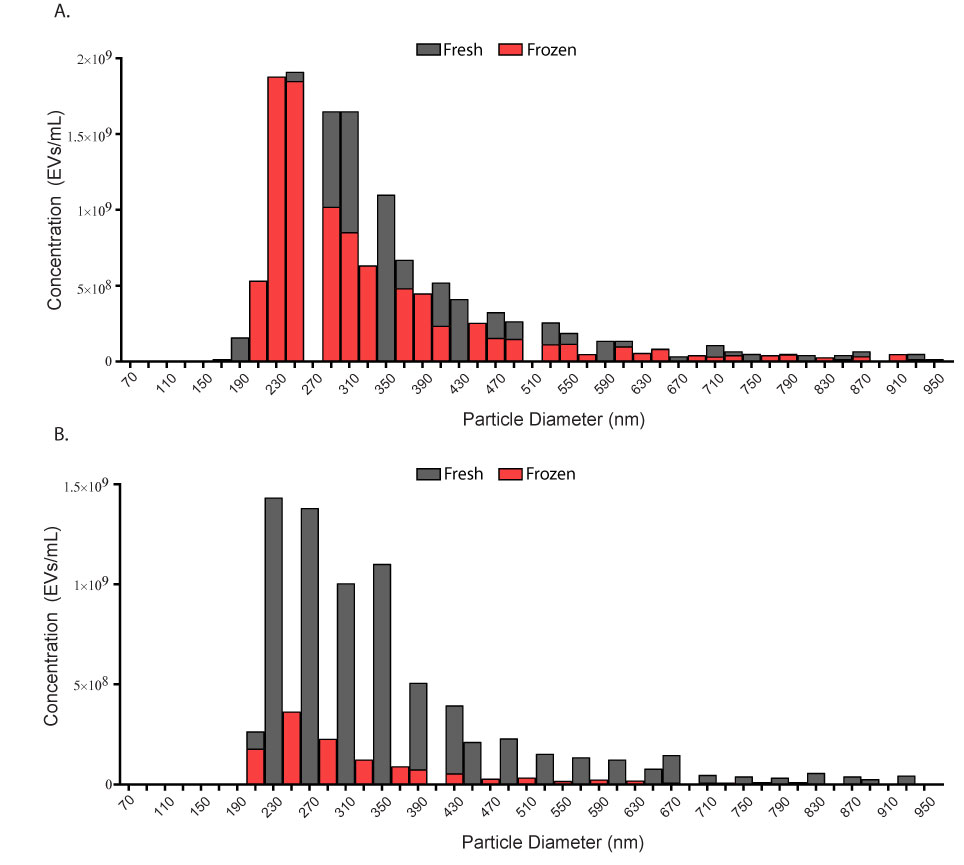


**Supplementary Figure 2**: *Impact of cryopreservation on particle diameter and size distribution of plasma-derived EVs*. All plasma samples were added directly to the -80°C freezer (without a freezing container), thawed at room temperature, and isolated using ultracentrifugation. (A-B) Representative histogram for two donors showing EV concentration binned by particle diameter (nm) with fresh samples shown in dark grey and stored samples in red.
