# Supplementary material for: Isolation of intact extracellular vesicles from cryopreserved samples
Source: PLoS One. 2021 May 13;16(5):e0251290. doi: 10.1371/journal.pone.0251290 (PMC8118530; doi:10.1371/journal.pone.0251290)
Supplement: S3 Fig — All plasma samples were added directly to the -80°C freezer (without a freezing container) and thawed at room temperature. All individual data points (or biological replicates) consist of paired fresh and stored samples that were obtained from the same individual in one blood draw. Solid lines between data points link the corresponding fresh and stored samples from a given individual. (A) RIN of EVs in stored versus fresh samples isolated using ultracentrifugation and extracted using the RNeasy spin columns are expressed as box-and-whiskers plots showing median, interquartile range, maxima/minima, and all individual data points (n = 10). (B-C) Representative RNeasy electropherograms showing RNA traces obtained from the Bioanalyzer. Fresh (black line) versus Stored (red line) denotes control (not frozen) versus samples frozen and thawed. (D) RIN of EVs in stored versus fresh samples isolated using ultracentrifugation and extracted using the miRNeasy spin columns are expressed as box-and-whiskers plots showing median, interquartile range, maxima/minima, and all individual data points (n = 9). (E-F) Representative miRNeasy electropherograms showing RNA traces obtained from the Bioanalyzer. Data were analyzed using the Wilcoxon signed-rank test (p<0.05); an asterisk indicates statistical significance. (DOCX) [file pone.0251290.s003.docx]

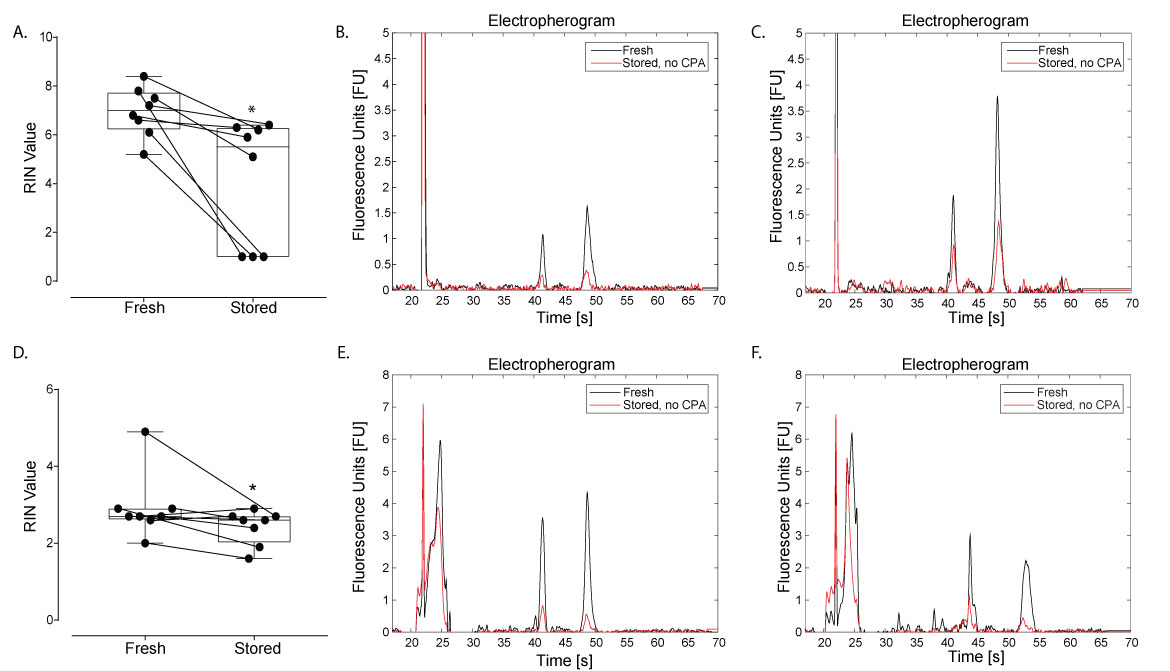


**Supplementary Figure 3**: *Impact of cryopreservation on RIN values of plasma-derived EVs*. All plasma samples were added directly to the -80°C freezer (without a freezing container) and thawed at room temperature. All individual data points (or biological replicates) consist of paired fresh and stored samples that were obtained from the same individual in one blood draw. Solid lines between data points link the corresponding fresh and stored samples from a given individual. (A) RIN of EVs in stored versus fresh samples isolated using ultracentrifugation and extracted using the RNeasy spin columns are expressed as box-and-whiskers plots showing median, interquartile range, maxima/minima, and all individual data points (n=10). (B-C) Representative RNeasy electropherograms showing RNA traces obtained from the Bioanalyzer. Fresh (black line) versus Stored (red line) denotes control (not frozen) versus samples frozen and thawed. (D) RIN of EVs in stored versus fresh samples isolated using ultracentrifugation and extracted using the miRNeasy spin columns are expressed as box-and-whiskers plots showing median, interquartile range, maxima/minima, and all individual data points (n=9). (E-F) Representative miRNeasy electropherograms showing RNA traces obtained from the Bioanalyzer. Data were analyzed using the Wilcoxon signed-rank test (p<0.05); an asterisk indicates statistical significance.
