# Supplementary material for: Isolation of intact extracellular vesicles from cryopreserved samples
Source: PLoS One. 2021 May 13;16(5):e0251290. doi: 10.1371/journal.pone.0251290 (PMC8118530; doi:10.1371/journal.pone.0251290)
Supplement: S4 Fig — (A) RNA Yield of EVs isolated from plasma using ultracentrifugation and treated with RNase A are expressed as box-and-whiskers plots showing median, interquartile range, maxima/minima, and all individual data points (n = 6). (B) RNA Yield of EVs isolated from media using qEV Columns and treated with RNase A are expressed as box-and-whiskers plots showing median, interquartile range, maxima/minima, and all individual data points (n = 4). (C) Positive immunoblot staining for CD9 for EVs isolated from plasma and media. Data were analyzed using the Mann-Whitney U-test (p<0.05); an asterisk indicates statistical significance. (DOCX) [file pone.0251290.s004.docx]

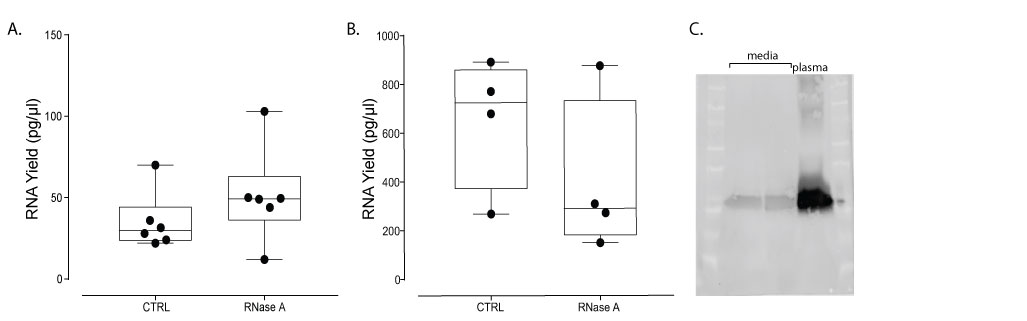


**Supplementary Figure 4**: *RNase A treatment and immunoblots of EVs isolated from plasma and media*. (A) RNA Yield of EVs isolated from plasma using ultracentrifugation and treated with RNase A are expressed as box-and-whiskers plots showing median, interquartile range, maxima/minima, and all individual data points (n=6). (B) RNA Yield of EVs isolated from media using qEV Columns and treated with RNase A are expressed as box-and-whiskers plots showing median, interquartile range, maxima/minima, and all individual data points (n=4). (C) Positive immunoblot staining for CD9 for EVs isolated from plasma and media. Data were analyzed using the Mann-Whitney U-test (p<0.05); an asterisk indicates statistical significance.
