# Supplementary material for: Isolation of intact extracellular vesicles from cryopreserved samples
Source: PLoS One. 2021 May 13;16(5):e0251290. doi: 10.1371/journal.pone.0251290 (PMC8118530; doi:10.1371/journal.pone.0251290)
Supplement: S1 Raw images — (PDF) [file pone.0251290.s005.pdf]

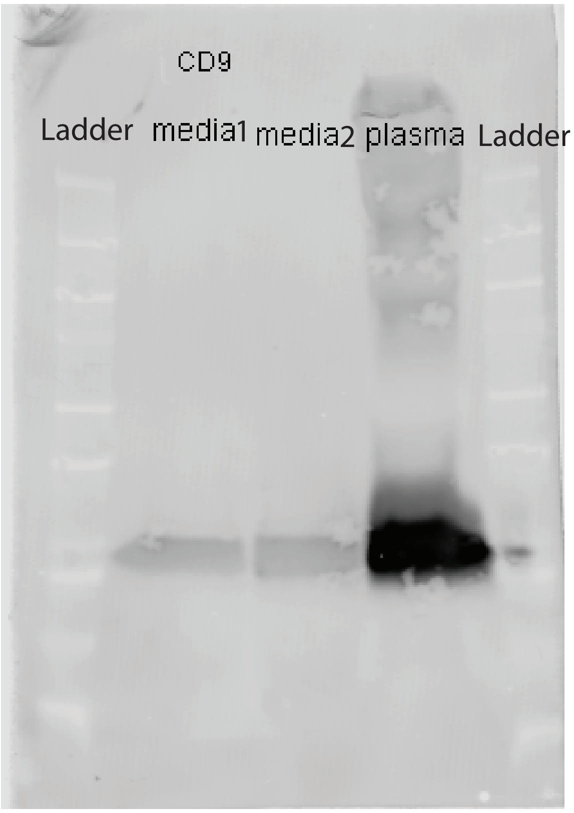

Raw immunoblot image for supplemental figure 4C

A 4-20% TGX gel was loaded as follows:
1) MW Ladder
2) BM1 Media qEV enriched EVs in non-reducing buffer (media1)
3) BM1 Media qEV enriched EVs in reducing buffer (media2)
4) Plasma EVs post ultracentrifugation (plasma)
5) MW Ladder

Lanes 1-5 were blotted for CD9

Following incubation with secondary antibodies the blot was imaged on a Licor Oddysey CLx imager
