# Supplementary material for: Isolation of intact extracellular vesicles from cryopreserved samples
Source: PLoS One. 2021 May 13;16(5):e0251290. doi: 10.1371/journal.pone.0251290 (PMC8118530; doi:10.1371/journal.pone.0251290)
Supplement: Attachment — Submitted filename: PLOSONE_ResponseReviewers_NewFinal.docx [file pone.0251290.s006.docx]

**Reviewer #1: Response to the Reviewer**

**This manuscript addresses the negative effects that cryopreservation may have on extracellular vesicles. The effect of two different cooling regimes, thawing regimes, storage periods, and RNA isolation kits, in addition of addition of the cryopreservation agent DMSO, on EV number, RNA yield and RNA quality was observed. Overall the subject of the study is most interesting and the results should be useful for researchers in the EV field. I do have some concerns, however, that I think warrant a major revision of the manuscript. These comments apply in particular to the presentation of the results and the thoroughness of the Materials and Methods section.**

We thank the reviewer for careful consideration of our manuscript, and we value the constructive feedback of our article. We address your specific comments below.

**1. Figures**

**Q1a. The figures are not all easy to interpret. For example, in Figure 2A, what is really Stored:Fresh? And what comprises one data point? Samples from the same donor? I think the presentation you use in Figure 2B should be included for all your figures, i.e., separate columns for fresh and stored samples. And it is quite unclear what the n value corresponds to. This should be described more clearly in the Materials and Methods section.**

Response Q1a: We have changed all figures, as requested, and all data is now presented as separate columns for Fresh and Stored. All individual data points (or biological replicates) consist of paired fresh and stored samples that were obtained from the same individual in one blood draw (for plasma) or same cell culture plate on the same day (for media). To try and emphasize/clarify this in the figure, we have also drawn solid lines between data points to link the corresponding fresh and stored samples from a given individual.

We have modified the section *Quantification and statistics* within the Materials and Methods to more clearly describe how data points are defined. It reads as follows:

“Each biological replicate from plasma represents one individual whereby the same blood draw from the same day was divided into paired fresh and stored samples of equal volume. Similarly, biological replicates for media samples are taken from the same cell culture plate from the same day and divided into paired fresh and stored samples of equal volumes. In all figures, solid lines between data points link the corresponding fresh and stored samples from a biological replicate.”

**Q1b. In the manuscript text you write that the plasma-derived EV concentration of stored samples was only 38% of fresh, non-frozen samples. When I look at Figure 2C, it appears as though the particle concentration at all particle diameters is much lower than this. If the figure cannot be interpreted as such, please explain.**

Response Q1b: We have made substantial changes to the figures based on this question and others (please also see response to Q1a). Figure 2c is data from one biological replicate and is not meant to represent the average across all biological replicates. Each biological replicate represents paired samples (one fresh and one stored) from one single individual obtained from a single blood draw on the same day. We have separated the fresh and stored samples into two columns, connected the paired samples with a solid line, and finally added language to the figure title and materials and methods to clarify. Taken together, we hope these changes enable easier interpretation of Figure 2c and d.

**Q1c. You keep referring to D0 and D10 in the figure legends while these annotations are nowhere to be found (only in the supplementary figures).**

Response Q1c: Our apologies for the confusion. The D0 and D10 annotations were used in previous versions of the manuscript prior to submission. We had decided to change D0 and D10 to fresh and stored, respectively, for the final submission since we thought it was clearer and we forgot to delete those annotations in the figure title. We have removed those annotations and clarified in the figure titles where appropriate. We have also fixed the annotations in the supplementary figures.

**Q1d. You never explain to the reader what an electropherogram depicts (you mention it very briefly in the Discussion, but that is not sufficient).**

Response Q1d: We have added content to the Materials and Methods section *RNA isolation and characterization* that describes what an electropherograms depicts.

The added content is provided below for reference:

“The Bioanalyzer uses capillary electrophoresis to assess RNA sizing, quantity, and integrity prior to downstream analysis. A fluorescent dye molecule intercalates into the RNA strand and bands are detected by their fluorescence. Bands are translated into both a gel-like image and peaks in the electropherogram. Each peak in the electropherogram is quantified in fluorescence units (y-axis) as a function of time (x-axis) since the speed in which RNA fragments move through the gel is proportional to their size. As such, RNA peaks closer to 20 seconds are smaller as compared to those that approach 70 seconds.”

**Q1e. In Figure 4, please chose a color other than blue. It is not that easy to distinguish it from the black line.**

Response Q1e: We have changed the color from blue to a dark yellow. We hope this is easier to distinguish from the black line.

**2. Materials and Methods, this section is generally lacking information. Please be more thorough, as suggested below:**

**Q2a. line 157: Add a comment that the method is described below**

Response Q2a: We have modified the sentence as follows (edits are highlighted in yellow):

“Fresh samples were then processed either by ultracentrifugation or using qEV Columns (Izon Science), followed by either particle analysis or RNA extraction (RNA concentration ranged from 17-1300 pg/µL), please see corresponding sections below that elaborate on each of these methods (i.e. *EV isolation*, *EV quantification*, and *RNA isolation and characterization*).”

**Q2b. line 158: Please explain the rationale for choosing 10-12 days. This amount of days seems in many respects meaningless low. In my own personal experience we normally have to store the EVs for much longer than that. I would think that holds true in many laboratories. While it certainly would have been impractical to wait for years (although I hope you stored some of your samples for a future study), you could have chosen the middle road and selected a few months.**

Response Q2b: We thank the reviewer for the opportunity to clarify. When we were designing this experiment originally, we had plans to look at both the impact of freeze-thaw versus short storage durations on the order of weeks versus longer durations on the order of years. However, there are a couple factors which influenced our decision to focus only on relatively short durations for this first paper.

Firstly, one very important feature of this paper in comparison to existing literature on the effect of EV and RNA yield during cryopreservation is the desire to have matched fresh controls for every biological replicate (e.g. each blood draw has a paired fresh and stored sample form the same donor). This is because donors can vary in the absolute number of EV, and this donor-to-donor variability is difficult to resolve against the effect of cryopreservation without matched controls. As a result, it was not possible to isolate enough EVs from one blood draw to perform a complete time series AND we aimed to have a relatively high number of replicates. Taken together, the experimental design was exceptionally laborious, and we had to choose one storage duration to compare with paired fresh controls.

We also address the second part of your question re: why we choose 12 days (~2 weeks) versus months versus years. As the reviewer notes, years is impractical. Our initial experiments started with weeks (i.e. up to 12 days) for two main reasons. Firstly, it gave us the opportunity to ascertain if there was any support for our hypothesis in a relatively short turnaround time. More importantly, we argued that if weeks showed significant degradation then it could be inferred that months of storage was at least equal (or worse). In contrast, if we stored for months, we would not know if samples would be okay for weeks. As such, we thought it made sense to start with shorter durations and build to longer storage durations in subsequent studies.

**Q2c. line 161: For how long were the samples kept in the freezing container?**

Response Q2c: Samples were kept in the freezing container for 6 hours at -80°C. This is enough time for samples to reach and stabilize to temperatures of -80°C. This experimental detail has been added to the Materials and Methods (see *EV sample processing section*) and is also specified in the results section as well as the corresponding figure title.

**Q2d. line 164: How many hours?**

Response Q2d: For samples thawed at room temperature it generally took a couple hours, while samples thawed in a warm water bath took a couple minutes. These experimental details have been added to line 164.

**Q2e. line 167: How much 10% DMSO did you add?**

Response Q2e: We add 400 µl of DMSO to 3.6 mL plasma (4 mL total volume). Hence this is a 10% v/v DMSO solution. This has been clarified in the Materials and Methods.

**Q2f. line 175, starting with “For ultracentrifugation,…”: Please revise the sentence; there is a grammar issue here.**

Response Q2f: The sentence has been revised as follows: “Samples were first spun at 300 rcf for 10 minutes at 4°C to remove cell contamination. Then, the supernatant was centrifuged at 2000 rcf for 10 minutes at 4°C to remove cellular debris and large vesicles. Subsequently, the supernatant was collected and filtered through a 0.8µm filter.”

**Q2g. line 179: You mix verb tenses several places, including here. Stick to one tense.**

Response Q2g: We have corrected this and taken care throughout the manuscript to remain consistent.

**Q2h. line 192: What is the rationale for choosing these two kits? Please explain here or in the Introduction.**

Response Q2h: We have added an explanation for choosing two kits to the materials and methods. It reads as follows (edits highlighted in yellow): “RNA was extracted using either the Qiagen RNeasy Plus Micro Kit (cat#74034) or miRNeasy Mini Kit (cat#217004) to ensure the trends we observed were not specific to downstream handling. This is especially relevant since miRNeasy spin columns have a higher binding affinity for small RNAs, as compared to RNeasy kits and the presence or absence of small RNAs can impact RIN values.

**Q2i. line 204: Again, I am having trouble deciphering the exact number of samples in your different experiments. With n=4 it is troublesome to perform a t-test. Additionally, did you check for normality?**

Response Q2i: We hope the changes to the figures, as outlined in response Q1a and b, have clarified the exact number of samples used in our experiments. Additionally, we indicate the number of biological replicates throughout the results section and in the figure titles. We have consulted with a statistician, who is now included as an author on this paper for her contributions. She has suggested we use a non-parametric test. As a result, we have changed all statistical analysis and updated all p-values to reflect these changes.


3. Additional comments:
**Q3a.** **Check singular vs. plural verb forms, f.ex. in line 32.**

Response Q3a: We have checked for singular versus plural forms in this location and others.

**Q3b. Shouldn´t there be a space before listing the reference number in the text?**

Response Q3b: I am not sure if there should be a space, although I believe these errors will be resolved when the manuscript is converted to a proof.

**Q3c. Line 66-73: Cumbersome language; please revise.**

Response Q3c.: These lines have been revised.

**Q3d. Line 109: Which are “all experimental conditions”?**

Response Q3d: we should have used the term “frozen/stored” instead of “all experimental conditions.” This has been changed in the manuscript.

**Q3e. Line 111 starting with “Further, …”: It is unclear what you mean.**

Response Q3e: Often in the field of biopreservation, there is not one storage method/protocol that fits all needs. For example, there may be instances where EVs only need to be preserved for a couple hours or days versus truly long-term banking for years. We attempted to clarify this in the text.

**Q3f. Line 115: Specify that you mean drawing of blood.**

Response Q3f: we have removed this from the sentence.

**Q3g. Line 115: Please move “including liposomes”. As it stands, it reads that lipid bilayers are sensitive to liposomes.**

Response Q3g: this has been deleted.

**Q3h. Line 121: What do you mean by “biologics”?**

Response Q3h: “biologics” refers to any material produced from a living organism or containing components of living organisms.

**Q3i. Line 217: Please explain what particle rate signifies.**

Response Q3i: Particle rate is the rate at which particles are counted in the qNano. A faster particle rate indicates a higher concentration of EVs. This has been clarified in the figure title of Supplementary Figure 1 and in the Results section.

**Q3j. Line 236: This belongs in the Materials and Methods section.**

Response Q3j: this has been moved to the Materials and Methods section

**Q3k. Line 238, starting with “This is…”: This belongs in the Materials and Methods section.**

Response Q3k: this has been moved to the Materials and Methods section

**Q3l. There is no sense in reporting p-values to four decimals.**

Response Q3l: we had reduced to two decimal places

**Q3m. Line 247: Please explain the RIN value in the Material and Methods section. Not all readers will understand what it signifies.**

Response Q3m: This has been added to the Materials and Methods section.

**Q3n. Line 268: This belongs in the Materials and Methods section.**

Response Q3n: this was added to the Materials and Methods section

**Q3o. Line 271, starting with “Secondly, …”: A bit unclear. What do you mean by traverse in this respect?**

Response Q3o: the temperature zone between -15 and -60°C is well known to cause serious damage to cells during cryopreservation. It is often suggested that it is the process of traversing this temperature zone, rather than the final holding temperature, that is the major source of damage. We have clarified this in the text.

**Q3p. Line 286: “bucket”?**

Response Q3p: we have changed this to Nalgene freezing container.

**Q3q. Line 329: Please explain the issue of EV count vs. RNA yield a bit more closely.**

Response Q3q: We apologize, but we are not certain what the reviewer is asking. We have added more data to support an overall loss of EVs because of freezing (see response to Q3r below). As a result, we have modified the language in this location to reflect those changes.

**Q3r. Line 337: Please discuss if your results could have been affected by the rather harsh ultracentrifugation process. Ideally, you should have used an additional EV isolation method.**

Response Q3r: We thank the reviewer for the comment and have added an additional EV isolation method (i.e. qEV Columns from Izon Science) to further strengthen the manuscript. Using the qEV Columns, we observed a trend indicative of decreasing RNA yield due to freezing, although this did not reach statistical significance due to a relatively low sample size (p=0.063, n=5). Due to challenges working with COVID restrictions, we were not able to complete additional replicates in time for the due date for this revised manuscript. We would welcome the opportunity to add more replicates for these qEV Column experiments to meet the rigor of our plasma experiments.

**Q3s. Line 357: This explanation should have been included in the Materials and Methods section. Same for line 363 (slow vs. fast).**

Response Q3s: We have added a more in-depth explanation about electropherograms/RIN values and their interpretation in the Materials and Methods section.

**Q3t. Line 374: Sentence unclear. Please revise.**

Response Q3t: This sentence was removed.

**Q3u. Line 387: Please explain glassy state/glass transition some more.**

Response Q3u: Freezing of a sample during cryopreservation results in the formation of an ice fraction and non-frozen fraction. This non-frozen fraction has a tendency to transition into the crystalline state, resulting in ice nucleation and crystal growth that can be very damaging for cells, EVs, or other biologics. To ensure this breakdown into the crystalline state does not occur, you typically aim to store samples below the glass transition temperature; however, typical plasma samples stored at -80°C are well above the glass transition temperature. DMSO is a well know “glass former” and by adding this cryoprotectant we raise the glass transition temperature and promote sample stability during cryopreservation. However, it should be noted that in this case, while DMSO does help promote stability, at the concentrations presently used, they are still not enough. Additional compounds with high glass transition temperatures can be supplemented to further promote stability during cryopreservation at -80°C. We have clarified this in the discussion.

**Q3v. Line 397: What do you mean with “While longer storage durations are required”?**

Response Q3v: We meant to say “While longer storage durations still need to be comprehensively studied…” This has been changed in the Discussion.

**Q3w. Line 457: The names of authors in reference 14 are all in capital letters.**

Response Q3w: This has been corrected.

**Reviewer #2: Response to Reviewer 2**

**Tessier et al. study the impact of a freeze thaw cycle of blood plasma on the integrity of EVs and their associated RNA cargo. Blood plasma was collected from healthy donors and stored at -80°C for 10-12 days in the presence or absence of cryoprotectant (DMSO) versus freshly processed. Stored samples were frozen directly or through means of freezing containers; and thawed at room temperature versus 37°C. EVs were separated from fresh or stored blood plasma by differential ultracentrifugation. Particle distributions were obtained by qNano measurements on EV preparations. In addition, RNA was isolated and yield was quantified. This study aims to contribute towards setting standards for blood processing for downstream EV analysis. Although of broad interest for the EV research field, there are several concerns.**

We thank the reviewer for careful consideration of our manuscript, and we value the constructive feedback of our article. We address your specific comments below.

**Q1)** **Since the authors separate EVs from blood plasma by differential ultracentrifugation the EV preparations are contaminated with other extracellular particles including RNA containing ribonucleoprotein complexes and lipoprotein particles. qNano cannot distinguish different types of extracellular particles. Thus, from the qNano experiments it is impossible to conclude that the decrease in particle concentration is attributed to the loss of EVs rather than to the loss of other extracellular particles (or a combined effect). AND Q2) The same holds true for the observed decrease in RNA yield. Is the decreased yield due to disruption of EVs or other extracellular particles? Did the authors measure RNA yield on total blood plasma, prior to EV separation? Would the authors observe the same losses if EV preparations from both fresh blood plasma and cryopreserved blood plasma were treated with a combination or not of RNase, protease and detergent? AND Q3) Comment 1 and 2 also account for testing the use of freezing containers, thawing at 37°C and adding DMSO to blood plasma prior to storage.**

Response Q1-3: We agree with the reviewer and because of these comments we have added more data, as follows:

a) We tested fresh versus frozen media samples that are devoid of some of the contaminating particles mentioned by the reviewer, including lipoprotein particles (new Figure 3c).
b) Further, we used qEV Columns as the isolation method for media samples, instead of ultracentrifugation, since these should also remove contaminating particles (new Figure 3c). Using the qEV Columns, we observed a trend indicative of decreasing RNA yield due to freezing, although this did not reach statistical significance due to a low sample size (p=0.063, n=5). Due to challenges working with COVID restrictions, we were not able to complete additional replicates in time for the due date for this revised manuscript. We would welcome the opportunity to add more replicates for these qEV Column experiments to meet the rigor of our plasma experiments.
c) Finally, we performed an RNase treatment on plasma- and media-derived EVs to ensure that loses in RNA were from intra-EV cargo (Supplementary Figure 4a-b). We observed no statistically significant difference between control and RNase A treated EVs with respect to RNA Yield.

**Q4) From the experiments presented in the manuscript it cannot be concluded that the observed decrease in number of particles and RNA yield can be attributed to EVs. Additional experiments should be performed including:
-assessing RNA yield in total blood plasma (prior to EV separation)
-assessing RNA and particle yield after RNase and/or protease and/or detergent treatment
-performing further characterization of EV preparations by electron microscopy and protein analysis (EV and non-EV associated proteins) to assess the composition of EV preparations (cfr MISEV2018 guidelines)**

Response Q4: Instead of assessing RNA yield in total blood plasma, we decided to use media and qEV Columns, as described above, to ensure that RNA loss was from EVs and not other particles. Further, we performed RNAse treatments and also performed immunoblots against CD9 on EV preparations isolated from plasma and media (Supplementary Figure 4).

**5) An alternative strategy that can be implemented is by the spiking an EV reference material, as is recently increasingly reported in literature, in blood plasma prior to testing different storage and thawing conditions. If not possible to include in the current experimental design of the study, it can be discussed by the authors.**

Response Q5: We have added the following statement as a future direction in the discussion: “In particular, future studies that spike in EV reference material in plasma and testing different storage and thawing conditions would be an area of immediate interest.”
