# Supplementary material for: Isolation of intact extracellular vesicles from cryopreserved samples
Source: PLoS One. 2021 May 13;16(5):e0251290. doi: 10.1371/journal.pone.0251290 (PMC8118530; doi:10.1371/journal.pone.0251290)
Supplement: Attachment — Submitted filename: PLOSONE_ResponseReviewers.docx [file pone.0251290.s008.docx]

**Reviewer #1: Response to the Reviewer**

We thank the reviewer for careful consideration and additional comments to further improve the manuscript. We address your specific comments below.

**Question 1:** Line 43: More common to write this as 47-52%.

**Response 1:** We have modified line 43 to read 47-52%, instead of 52-47%.

**Question 2:** Line 81/112: Specify which media you refer to (cell culture supernatants? Or does it refer to the media in the section starting with 157?).

**Response 2**: We have changed line 81 and 112 to read “cell culture supernatants,” instead of media.

**Question 3:** Line 157: This section is missing some information (BM1 palm-tdTomato cells? Not easy to understand for those who are not used to working with your material).

**Response 3**: We have added information about the BM1- palm-tdTomato cells to the Materials and Methods section as well as included references which detail how they were generated. These changes are as follows (changes are highlighted in yellow): “BM1 cells are a highly invasive bone-metastatic variant of MDA-MB-231 cells (the most used cell line to study triple negative breast cancer) that generate a lot of EVs. To generate fluorescent EV reporters for direct visualization of cargo, a palmitoylation signal was genetically fused to the N-terminus of tdTomato [32,33].”

**Question 4**: Could storage in a DMSO-containing solution impact later downstream analysis of f.ex. EV content? I believe the presence of DMSO can change fluxes of ions and molecules through cellular plasma membranes.

**Response 4:** this is a great question from the reviewer that is certainly important. DMSO should be removed during EV isolation, including ultracentrifugation and size exclusion chromatography, like the IZON qEV columns used in the present manuscript. Other common methods not used in the present manuscript to isolate EVs, including polymer-based precipitations, density gradient centrifugation and microfluidics isolation technology, should also remove DMSO during EV isolation. We have modified this sentence in the discussion to make this point (changes are highlighted in yellow): “Further, we describe how the addition of the cryoprotectant agent, DMSO, can overcome some forms of cryoinjury, although longer-term preservation studies are a necessary next step. Importantly, this method is relatively easy, does not require any expensive equipment, and is compatible with downstream assays since DMSO should be removed during isolation, making it feasible for research and clinical implementation.”
