# Supplementary material for: Isolation of intact extracellular vesicles from cryopreserved samples
Source: PLoS One. 2021 May 13;16(5):e0251290. doi: 10.1371/journal.pone.0251290 (PMC8118530; doi:10.1371/journal.pone.0251290)
Supplement: Attachment — Submitted filename: reviewer comments.docx [file pone.0251290.s007.docx]

Isolation of intact extracellular vesicles from cryopreserved samples by Shannon N. Tessier, Lauren D. Bookstaver, Cindy Angpraseuth, Cleo J. Stannard, Beatriz Marques, Uyen K. Ho1, Alona Muzikansky, Berent Aldikacti, Eduardo Reátegui, Daniel C. Rabe, Mehmet Toner, and Shannon L. Stott.

The manuscript has been significantly improved and the authors have addressed all my comments. I only have these few items I´d like to address:

Line 43: More common to write this as 47-52%.

Line 81/112: Specify which media you refer to (cell culture supernatants? Or does it refer to the media in the section starting with 157?).

Line 157: This section is missing some information (BM1 palm-tdTomato cells? Not easy to understand for those who are not used to working with your material).

Last note: Could storage in a DMSO-containing solution impact later downstream analysis of f.ex. EV content? I believe the presence of DMSO can change fluxes of ions and molecules through cellular plasma membranes.
